# Optimization of norepinephrine dosage for hemodynamic stability during ECMO support in refractory shock: a retrospective study

**DOI:** 10.3389/fmed.2026.1804565

**Published:** 2026-04-29

**Authors:** Lele Huang, Yajun Li, Zhenzhen Chen

**Affiliations:** Department of Critical Care Medicine, The First Affiliated Hospital of Bengbu Medical University, Bengbu, China

**Keywords:** extracorporeal membrane oxygenation, hemodynamics, mortality, norepinephrine, refractory shock

## Abstract

**Background:**

Veno-arterial extracorporeal membrane oxygenation (VA-ECMO) serves as a crucial salvage therapy for refractory shock; however, the optimal strategy for vasopressor management during extracorporeal circulatory support remains unclear. Norepinephrine is a cornerstone agent for maintaining perfusion pressure, yet the association between its dose intensity and patient outcomes remains controversial.

**Objective:**

This study aimed to investigate the association between the norepinephrine-equivalent dose during the initial phase of VA-ECMO support and clinical outcomes in patients with refractory shock.

**Methods:**

In this retrospective cohort study, 135 patients with refractory shock supported by VA-ECMO in the intensive care unit (ICU) of our hospital from January 2020 to January 2025 were included. Patients were divided into a low-dose group (*n* = 68) and a high-dose group (*n* = 67) based on the median value of the mean norepinephrine-equivalent dose within 72 h after ECMO initiation. Baseline characteristics, 30-day all-cause mortality, a composite endpoint of hemodynamic stability (proportion of time meeting target thresholds between 24 and 72 h of ECMO support), successful ECMO weaning rate, ICU length of stay, total duration of vasopressor support, and indicators of tissue perfusion and organ function [24 h lactate clearance, *Δ* Sequential Organ Failure Assessment (SOFA) score, and the incidence of acute kidney injury] were compared between groups.

**Results:**

At baseline, the high-dose group had significantly higher SOFA scores (*t* = 4.156, *p* < 0.001) and serum lactate levels (*t* = 4.893, *p* < 0.001) compared to the low-dose group. Survival analysis demonstrated a significantly lower 30-day cumulative survival rate in the high-dose group (log-rank χ^2^ = 9.873, *p* = 0.002). After adjustment using multivariable Cox regression, a high vasopressor dose remained an independent risk factor for mortality (HR = 1.92, 95% CI 1.13–3.25, *p* = 0.015). Regarding secondary outcomes, the proportion of patients achieving hemodynamic stability was significantly lower in the high-dose group (χ^2^ = 10.897, *p* = 0.001). This group also exhibited a lower successful ECMO weaning rate (χ^2^ = 8.352, *p* = 0.004), a longer ICU stay (*t* = 2.341, *p* = 0.021), and a prolonged duration of vasopressor support (*t* = 3.894, *p* < 0.001). Tissue perfusion metrics revealed that the 24 h lactate clearance rate was lower in the high-dose group (*t* = −3.125, *p* = 0.002), and the improvement in SOFA score at 72 h was worse (*t* = 2.894, *p* = 0.004). Furthermore, the incidence of acute kidney injury (χ^2^ = 4.012, *p* = 0.045) and the new-onset severe arrhythmias (χ^2^ = 5.124, *p* = 0.024) were higher in the high-dose group.

**Conclusion:**

Among patients with refractory shock supported by VA-ECMO, a higher norepinephrine-equivalent dose during the initial support period is independently associated with an increased risk of death and worse clinical outcomes. This suggests the need for careful evaluation and optimization of vasopressor intensity, even when ECMO provides circulatory support.

## 1 Introduction

Refractory shock represents a profoundly challenging clinical syndrome in critical care medicine, characterized by persistent hypotension and tissue hypoperfusion despite adequate fluid resuscitation, and its pathological trajectory often proves resistant to conventional vasoactive drug therapy (1). As an ultimate modality for circulatory and respiratory support, veno-arterial extracorporeal membrane oxygenation (VA-ECMO) can provide temporary life support for such patients, buying valuable time for definitive treatment of the underlying cause and recovery of organ function (2). However, even with the robust circulatory support afforded by ECMO, most patients require concomitant administration of vasopressors, particularly norepinephrine, to maintain necessary perfusion pressure and optimize organ blood flow distribution (3). Norepinephrine, an *α*-adrenergic receptor agonist, increases mean arterial pressure via vasoconstriction, yet the relationship between its dose and clinical outcomes remains far from clear in complex, critically ill populations (4). In the setting of shock, excessive catecholamine release combined with high-dose exogenous infusion may precipitate a range of potential detriments, including the exacerbation of microcirculatory dysfunction, increased myocardial oxygen consumption, the provocation of arrhythmias, and potential immune dysregulation (5). Therefore, within the unique context of “partial circulatory replacement” provided by ECMO, achieving a balance between the pressor effects of vasoactive drugs and their potential toxicity to enable refined hemodynamic management is a pivotal clinical dilemma (6).

Despite the increasing adoption of ECMO technology, high-quality evidence guiding optimal vasopressor strategies during support remains scarce (7). Existing clinical research has predominantly focused on ECMO cannulation timing, anticoagulation management, or complication prevention, with less attention paid to the synergistic pharmacological management atop the provided circulatory support (8). Many clinicians adjust drug doses based on experience or guidelines derived from non-ECMO shock patients, an approach that may not be appropriate for individuals whose hemodynamic milieu has been fundamentally altered by ECMO support (9). Observational studies have suggested an association between high-dose vasopressors and adverse outcomes in ECMO patients; however, these investigations have often failed to adequately adjust for the substantial differences in baseline illness severity (10). For instance, sicker patients inherently require higher drug support, which can create a spurious association between “dose” and “death” (11). Moreover, prior studies frequently prioritized blood pressure as a core target, potentially overlooking more crucial endpoints of tissue perfusion, such as lactate clearance and organ function evolution (12). There is a paucity of research specifically targeting the critical initial time window of ECMO support to systematically evaluate the association between norepinephrine dose intensity and composite clinical outcomes encompassing hemodynamic stability, organ function recovery, and ultimate survival (13). This knowledge gap contributes to significant heterogeneity in clinical practice, ranging from aggressive use aimed at rapidly achieving target blood pressure to conservative strategies advocating for the “lowest effective dose” (14). Clarifying this association is crucial for developing standardized, evidence-based hemodynamic management protocols (15).

Accordingly, this study aims to conduct a retrospective cohort analysis to investigate the association between the intensity of norepinephrine-equivalent dose during the first 72 h following VA-ECMO support and clinical prognosis in patients with refractory shock. While all patients with refractory shock, by definition, receive substantial vasopressor support, we hypothesized that even within this uniformly high-risk population, there exists a dose-dependent association between vasopressor intensity and adverse outcomes. Specifically, we sought to determine whether patients receiving higher vasopressor doses (above the cohort median) would demonstrate worse clinical outcomes, including mortality, hemodynamic stability, and organ function recovery, after accounting for baseline illness severity. We focus not only on traditional survival outcomes but also introduce composite indicators reflecting the quality of hemodynamic stability, improvement in tissue perfusion, and treatment burden. By employing multivariable modeling and methods such as propensity score matching, we aim to control for confounding effects arising from baseline illness severity, providing more robust clinical evidence to guide the precise use of vasoactive drugs during ECMO support.

## 2 Materials and methods

### 2.1 General information

This study was designed as a single-center, retrospective cohort study conducted in the Intensive Care Unit (ICU) of our hospital. We consecutively enrolled adult patients (≥18 years) with refractory shock who received VA-ECMO support from January 2020 to January 2025. The exposure variable was defined as the mean norepinephrine-equivalent dose during the initial 72 h following ECMO support initiation (detailed below). Group stratification was based on the median value of this dose across the entire cohort. Specifically, patients were categorized into a “low vasopressor dose group” (dose ≤ median) and a “high vasopressor dose group” (dose > median). In this study, all patients meeting the inclusion and exclusion criteria were included in the analysis, resulting in a final cohort of 135 patients: 68 in the low-dose group and 67 in the high-dose group. This approximate 1:1 group ratio reflects the natural distribution of vasopressor requirements in clinical practice.

The sample size was not determined based on a pre-specified power calculation but included all consecutive eligible cases from our ECMO center database over the 5-year period. A *post-hoc* power analysis was performed to assess the reliability of the findings. Setting a significance level (*α*) of 0.05 (two-sided) and assuming detection of a 20% absolute difference in 30-day all-cause mortality (e.g., 40% in the low-dose group vs. 60% in the high-dose group) as a clinically relevant effect size, a *post-hoc* power analysis for the log-rank test based on the total sample of 135 and the observed event rates indicated a statistical power exceeding 80%. This suggests that the current sample size was sufficient to detect the pre-specified clinically significant difference.

### 2.2 Inclusion and exclusion criteria

Patient selection strictly adhered to the following criteria:

Inclusion Criteria:

1. Age ≥ 18 years.
2. Clinical diagnosis of refractory shock, specifically defined as: persistent hypotension (mean arterial pressure < 65 mmHg) accompanied by signs of tissue hypoperfusion (e.g., lactate > 2.0 mmol/L, skin mottling, oliguria) despite adequate fluid resuscitation and use of moderate-to-high dose vasoactive drugs (e.g., norepinephrine > 0.1 μg/kg/min). The underlying etiologies of refractory shock in the study cohort were predominantly cardiogenic [including acute myocardial infarction (54.8%) and fulminant myocarditis (23.7%)], with a smaller proportion of distributive shock secondary to severe sepsis or septic shock (21.5%).
3. Received VA-ECMO support as salvage therapy for the aforementioned refractory shock.
4. ECMO support duration of at least 72 h to ensure a sufficient time window for assessing hemodynamic response and to align with the norepinephrine-equivalent dose calculation window (first 72 h post-ECMO initiation).

Exclusion Criteria:

1. Presence of unequivocal irreversible brain injury or terminal illness with an expected survival of less than 24 h prior to ECMO initiation.
2. Pregnancy.
3. Critical clinical missing data exceeding 20%, including pre-ECMO vasopressor dose, baseline Sequential Organ Failure Assessment (SOFA) score, and hemodynamic and lactate data from the initial ECMO support period.
4. Patients receiving ECMO electively for scheduled cardiac surgery (e.g., heart transplantation, ventricular assist device implantation), as their clinical profile is fundamentally different from that of emergency salvage ECMO.

### 2.3 Research methods

1. Data Collection Process and Quality Control: All data were extracted from our hospital’s unified electronic medical record system (Hospital Information System, HIS) and Laboratory Information Management System (LIS). Data extraction was performed independently by two intensive care research fellows who were not involved in clinical decision-making and blinded to the study hypothesis. A detailed data extraction manual was developed, standardizing the definition, source, and extraction time point for each variable. Extracted data were entered into a pre-designed, encrypted electronic case report form. For data items with discrepancies between the two extractors, final values were determined through re-evaluation of the original medical records and arbitration by a third senior intensive care specialist. This blinded data extraction process was designed to minimize information bias.
2. Definition and Calculation of Exposure Variable (Norepinephrine Dose): The core exposure variable was defined as the mean norepinephrine-equivalent dose during the first 72 h after ECMO initiation. To unify the pressor effects of different vasoactive agents, the doses of these agents were converted to norepinephrine-equivalent doses. The pharmacological effects of dopamine are dose-dependent. In the clinical management of refractory shock, dopamine is typically administered at doses exceeding 5–10 μg/kg/min, at which *α*-adrenergic effects predominate and exert a clear vasopressor action. Based on this clinical pharmacological consensus, the following conversion formula was applied: norepinephrine dose remained unchanged; epinephrine was converted at a 1:1 ratio; dopamine (μg/kg/min) was divided by 2 for conversion; and vasopressin at 0.04 units/h was considered equivalent to norepinephrine 0.1 μg/kg/min. This conversion method has been widely adopted in previous clinical studies to standardize the intensity of different vasoactive agents. For each patient, the arithmetic mean of the norepinephrine-equivalent doses recorded at all-time points (typically hourly) from ECMO initiation (T0) to T72 was calculated and used as the “mean dose.” This continuous variable was used both for median-based grouping and as a continuous variable for subsequent dose–response relationship analysis.
3. Study Timeline and Key Time Points: Several key observation time points were defined for dynamic assessment: T0 (immediately at ECMO initiation), T24 (24 h post-ECMO initiation), T72 (72 h post-ECMO initiation), and T-endpoint (at ECMO decannulation or the last complete record prior to patient death). All time-dependent variables (e.g., vital signs, laboratory values, drug doses) were aligned with these predefined time points. For records not precisely at these times, the closest value within 1 h before or after the time point was taken.
4. Bias Control Strategies: To control for the influence of known confounders on outcomes, particularly the inherent imbalance in baseline illness severity between groups, two complementary statistical strategies were employed. First, in the primary analysis, a multivariable Cox proportional hazards regression model was used, pre-specifying the following clinically important confounders as covariates: age, SOFA score prior to ECMO initiation, serum lactate level prior to ECMO initiation, and primary etiology (acute myocardial infarction vs. fulminant myocarditis vs. other). The use of other vasoactive agents (epinephrine, dopamine, vasopressin) was accounted for by converting all agents to norepinephrine-equivalent doses, as described above. Second, as a sensitivity analysis to enhance the robustness of causal inference, propensity score matching (PSM) was performed. A logistic regression model based on the same confounders listed above was used to calculate a propensity score for each patient, representing their probability of belonging to the high-dose group. Subsequently, using 1:1 nearest-neighbor matching within a caliper width set to 0.02 times the standard deviation of the logit of the propensity score, each patient in the high-dose group was matched to one patient in the low-dose group with the closest propensity score. This yielded a sub-cohort with more balanced baseline characteristics for repeat analysis.

### 2.4 Outcome measures

(1) Primary Outcome Measure:

30-day all-cause mortality: Defined as death from any cause within 30 days from the date of VA-ECMO support initiation. Survival status was confirmed via hospital record systems and, when necessary, telephone follow-up.

(2) Key Secondary Outcome Measures:

Surrogate Indicator of Hemodynamic Stability: This indicator aimed to reflect the stability of macrocirculatory perfusion during the initial ECMO support phase. It was defined as the cumulative percentage of time during the 48 h observation period from T24 to T72 that a patient’s mean arterial pressure (MAP) was maintained within a preset target range (65–75 mmHg). While we acknowledge that an ideal composite endpoint would incorporate multiple physiological parameters, MAP represents a critical and widely used surrogate for adequate organ perfusion pressure in clinical practice. This single-component indicator was chosen to provide a standardized, objective measure of blood pressure control during the early ECMO period. This percentage was derived by calculating the proportion of hourly recorded MAP values falling within this range out of the total number of recordings. Stability was defined as achieving ≥70%, while <70% was considered unstable.

Successful ECMO Weaning Rate: An ECMO weaning attempt was defined as the process of gradually reducing ECMO flow to minimal levels (typically <1.0 L/min) and maintaining stability for several hours following improvement in hemodynamics and oxygenation. Successful weaning required meeting two criteria: (a) successful removal of the ECMO circuit, and (b) survival for at least 48 h post-decannulation without requiring re-initiation of ECMO support.

ICU Length of Stay: Calculated as the cumulative number of days from ICU admission to discharge from the ICU or death.

Total Duration of Vasopressor Support: Calculated as the total number of hours from the initiation of any vasoactive drug to the complete discontinuation of all vasoactive agents.

(3) Dynamic Indicators of Tissue Perfusion and Organ Function:

Lactate Clearance Rate: An indicator assessing early improvement in tissue perfusion. Calculated as: [(T0 lactate - T24 lactate)/T0 lactate] × 100%. A clearance rate ≥10% is typically considered clinically favorable.

Dynamic Change in SOFA Score: The SOFA score, quantifying organ dysfunction across six systems (respiratory, coagulation, hepatic, cardiovascular, neurological, renal), each scored 0–4 (total 0–24), was calculated at T0, T24, T72, and T-endpoint (if alive). ΔSOFA (e.g., T72 score - T0 score) was calculated to assess organ function evolution.

Incidence of Acute Kidney Injury (AKI): Diagnosed and staged according to Kidney Disease: Improving Global Outcomes (KDIGO) criteria, evaluating new-onset or worsening AKI during ECMO support.

Incidence of New-Onset Severe Arrhythmias: Documented new occurrences of tachyarrhythmias (e.g., ventricular tachycardia, ventricular fibrillation) or bradyarrhythmias during ECMO support requiring pharmacological or electrical intervention.

(4) Indicators of Treatment Intensity and Support Measures:

Total Days of Mechanical Ventilation: Calculated from initiation of invasive mechanical ventilation via endotracheal intubation to successful extubation or death.

Utilization Rate of Continuous Renal Replacement Therapy (CRRT): Documented whether CRRT was administered during ECMO support for reasons such as AKI, severe electrolyte imbalance, or volume overload.

Total Packed Red Blood Cell Transfusion Volume: The total number of units of packed red blood cells transfused during the entire hospitalization (1 unit ≈ 200 mL of red cells), serving as a surrogate for bleeding and hemodilution.

(5) Safety and Complication Indicators:

Incidence of ECMO Limb Distal Ischemia: Ischemic events in the limb ipsilateral to the ECMO cannulation site, confirmed by clinical examination (pain, pallor, pulselessness, paresthesia, paralysis) and/or Doppler ultrasound.

Incidence of Major Bleeding Events: Defined as Bleeding Academic Research Consortium (BARC) type 3 (overt bleeding leading to a hemoglobin drop of 3–5 g/dL or requiring transfusion) or higher bleeding.

Incidence of Confirmed Hospital-Acquired Infections: Diagnosed according to Centers for Disease Control and Prevention (CDC) criteria, including new-onset ventilator-associated pneumonia, catheter-related bloodstream infection, or ECMO circuit-related infection during ECMO support.

The management of complications was guided by standard institutional protocols. For acute limb ischemia, management included placement of a distal perfusion catheter if not already in place, surgical thrombectomy (Fogarty procedure) when indicated, or, in severe cases with irreversible ischemia, consideration of early ECMO weaning and decannulation. The decision to discontinue ECMO support was based on overall clinical stability, evidence of cardiac recovery, and the feasibility of managing complications conservatively or interventionally. ECMO was not routinely discontinued solely due to the occurrence of limb ischemia or bleeding unless these complications were deemed life-threatening or unmanageable.

### 2.5 Statistical analysis

All statistical analyses were performed using the R language (version 4.3.0; R Foundation for Statistical Computing, Vienna, Austria) and relevant packages. Continuous variables were first tested for normality (Shapiro–Wilk test). Normally distributed data are presented as mean ± standard deviation, with between-group comparisons made using independent samples *t*-tests. Non-normally distributed data are presented as median (interquartile range), with between-group comparisons made using Mann–Whitney U tests. Categorical variables are presented as frequency (percentage), with between-group comparisons made using chi-square tests or Fisher’s exact tests (when expected frequencies were less than 5).

For the primary outcome of 30-day survival, Kaplan–Meier curves were plotted, and survival differences between groups were compared using the log-rank test. To assess the impact of norepinephrine dose on mortality risk while controlling for confounding, a multivariable Cox proportional hazards regression model was constructed. The core exposure variable (norepinephrine dose group) was entered as the primary predictor, with adjustment for covariates including age, baseline SOFA score, baseline lactate, and primary diagnosis. Model results are presented as adjusted hazard ratios with 95% confidence intervals. The proportional hazards assumption for the Cox model was verified using Schoenfeld residual tests.

To explore potential non-linear relationships between the norepinephrine dose (as a continuous variable) and 30-day mortality risk, restricted cubic spline models were fitted with 3 knots (at the 10th, 50th, and 90th percentiles) and visualized using the median dose as the reference point.

As described earlier, propensity score matching was performed as a sensitivity analysis. Baseline characteristics of the matched cohort were reassessed for balance. Subsequently, Kaplan–Meier survival analysis and Cox regression analysis (including only the matching variables as covariates) were repeated in the matched sample to test the robustness of the primary finding.

For all statistical tests, a *p* < 0.05 was considered statistically significant. Adjustments for multiple comparisons were applied where appropriate.

## 3 Results

### 3.1 Patient screening and baseline characteristics

A total of 182 patients who received VA-ECMO support between January 2020 and January 2025 were initially screened for this study. After applying strict inclusion and exclusion criteria, 135 patients were ultimately enrolled in the final analysis cohort. Based on the median value (0.28 μg/kg/min) of the mean norepinephrine-equivalent dose during the first 72 h post-ECMO initiation, patients were stratified into two groups: the low-dose group (≤0.28 μg/kg/min, *n* = 68) and the high-dose group (>0.28 μg/kg/min, *n* = 67).

**Figure 1:**
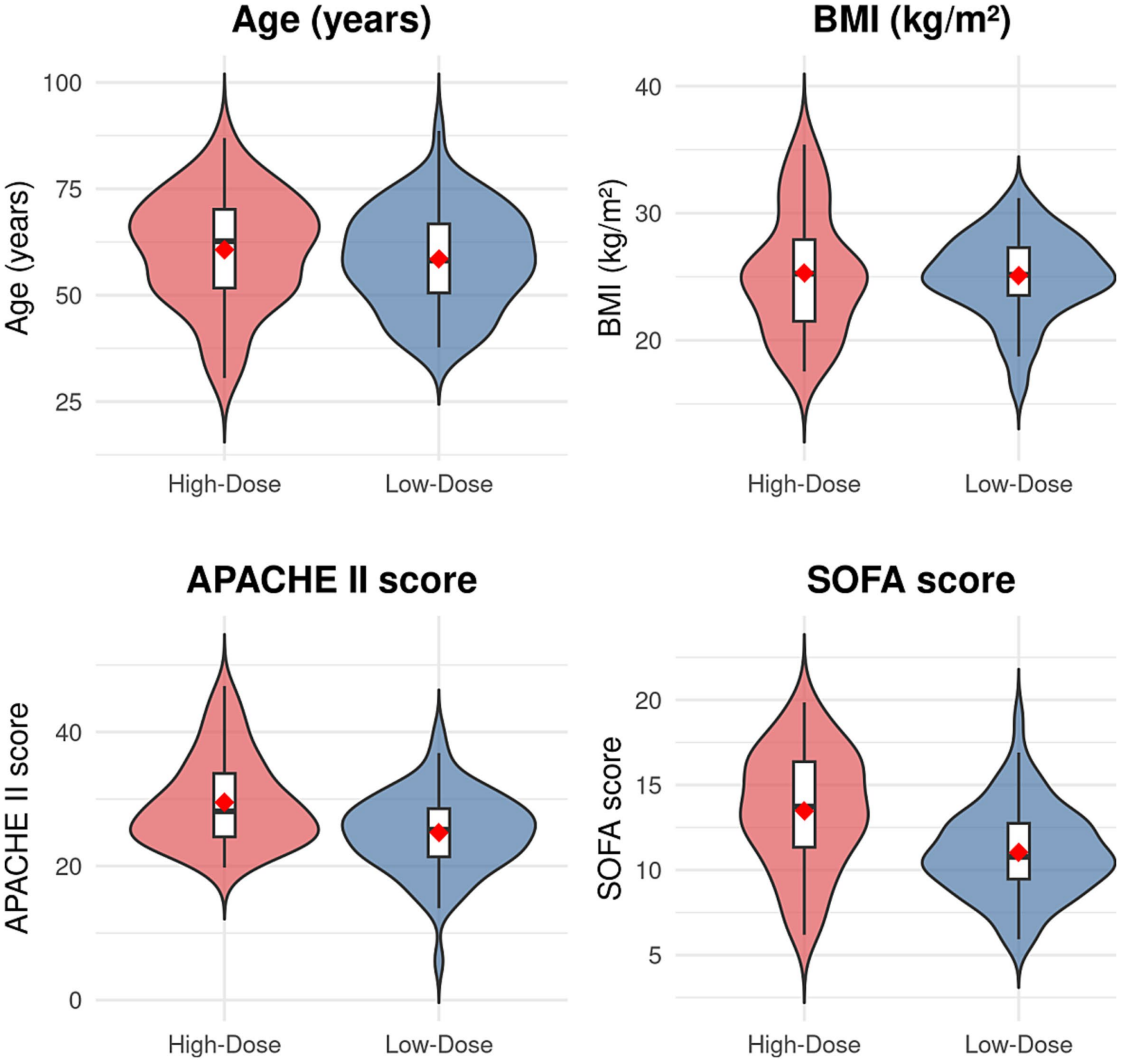
Baseline characteristics by treatment group.

A comparison of the baseline characteristics between the two groups is detailed in Table 1. The results indicated no statistically significant differences between the groups regarding demographic data (age, sex, BMI) or the distribution of primary diagnoses (acute myocardial infarction, fulminant myocarditis) (*p* > 0.05). However, markers of disease severity revealed significant disparities: patients in the high-dose group had significantly higher APACHE II and SOFA scores before ECMO implantation compared to the low-dose group (t = 3.217, *p* = 0.002; *t* = 4.892, *p* < 0.001). The high-dose group also exhibited worse hemodynamic status immediately prior to ECMO cannulation, manifested as a higher heart rate (*t* = 2.541, *p* = 0.012), a lower mean arterial pressure (*t* = −3.784, *p* < 0.001), and a higher baseline norepinephrine infusion rate (*t* = 5.632, *p* < 0.001). Regarding laboratory parameters, serum lactate and procalcitonin levels were significantly elevated in the high-dose group (*t* = 4.156, *p* < 0.001; *t* = 3.045, *p* = 0.003), while the left ventricular ejection fraction was significantly lower (*t* = −2.987, *p* = 0.003). The prevalence of comorbidities, including hypertension, coronary artery disease, diabetes mellitus, and chronic renal insufficiency, did not differ significantly between the groups (*p* > 0.05) (see Table 1 and Figure 1).

**Table 1:** Comparison of baseline characteristics between the low-dose and high-dose groups.

Characteristic	Low-dose group (*n* = 68)	High-dose group (*n* = 67)	Statistic	*P*-value
Demographics				
Age (years), mean ± SD	58.34 ± 12.67	60.18 ± 11.92	*t* = −0.875	0.383
Male, n (%)	45 (66.18)	48 (71.64)	χ^2^ = 0.476	0.49
BMI (kg/m^2^), mean ± SD	24.87 ± 3.56	25.41 ± 4.02	*t* = −0.832	0.407
Primary diagnosis, n (%)				
Acute myocardial infarction	38 (55.88)	36 (53.73)	χ^2^ = 0.067	0.795
Fulminant myocarditis	17 (25.00)	15 (22.39)	χ^2^ = 0.126	0.723
Illness severity scores				
APACHE II score, mean ± SD	25.63 ± 6.12	28.74 ± 7.05	*t* = −2.775	0.006
SOFA score, mean ± SD	11.24 ± 3.01	13.58 ± 3.47	*t* = −4.156	<0.001
Pre-ECMO status				
Heart rate (bpm), mean ± SD	112.45 ± 18.32	121.67 ± 20.13	*t* = −2.831	0.005
Mean arterial pressure (mmhg), mean ± SD	62.34 ± 8.76	57.12 ± 9.45	*t* = 3.347	0.001
Norepinephrine dose (μg/kg/min), mean ± SD	0.19 ± 0.08	0.35 ± 0.12	*t* = −9.257	<0.001
Lvef (%), mean ± sd	24.56 ± 7.83	20.12 ± 8.54	*t* = 3.087	0.002
Key laboratory values				
Serum creatinine (μmol/L), median (IQR)	132.5 (98.8–187.2)	158.3 (115.6–221.4)	*Z* = −1.874	0.061
Lactate (mmol/L), mean ± sd	5.67 ± 2.34	7.89 ± 3.01	*t* = −4.893	<0.001
Procalcitonin (ng/mL), median (IQR)	8.45 (3.12–25.60)	18.90 (5.44–52.10)	*Z* = −2.988	0.003
NT-probnp (pg/mL), median (IQR)	4,520 (2,150–9,850)	5,870 (3,120–12,400)	*Z* = −1.645	0.1
Comorbidities, n (%)				
Hypertension	32 (47.06)	35 (52.24)	χ^2^ = 0.369	0.544
Diabetes mellitus	18 (26.47)	22 (32.84)	χ^2^ = 0.683	0.409
Chronic renal insufficiency	9 (13.24)	13 (19.40)	χ^2^ = 0.958	0.328

BMI, body mass index; APACHE II, acute physiology and chronic health evaluation II; SOFA, sequential organ failure assessment; LVEF, left ventricular ejection fraction; IQR, interquartile range; NT-proBNP, N-terminal pro-B-type natriuretic peptide.

### 3.2 Primary outcome: 30-day survival analysis

**Figure 2:**
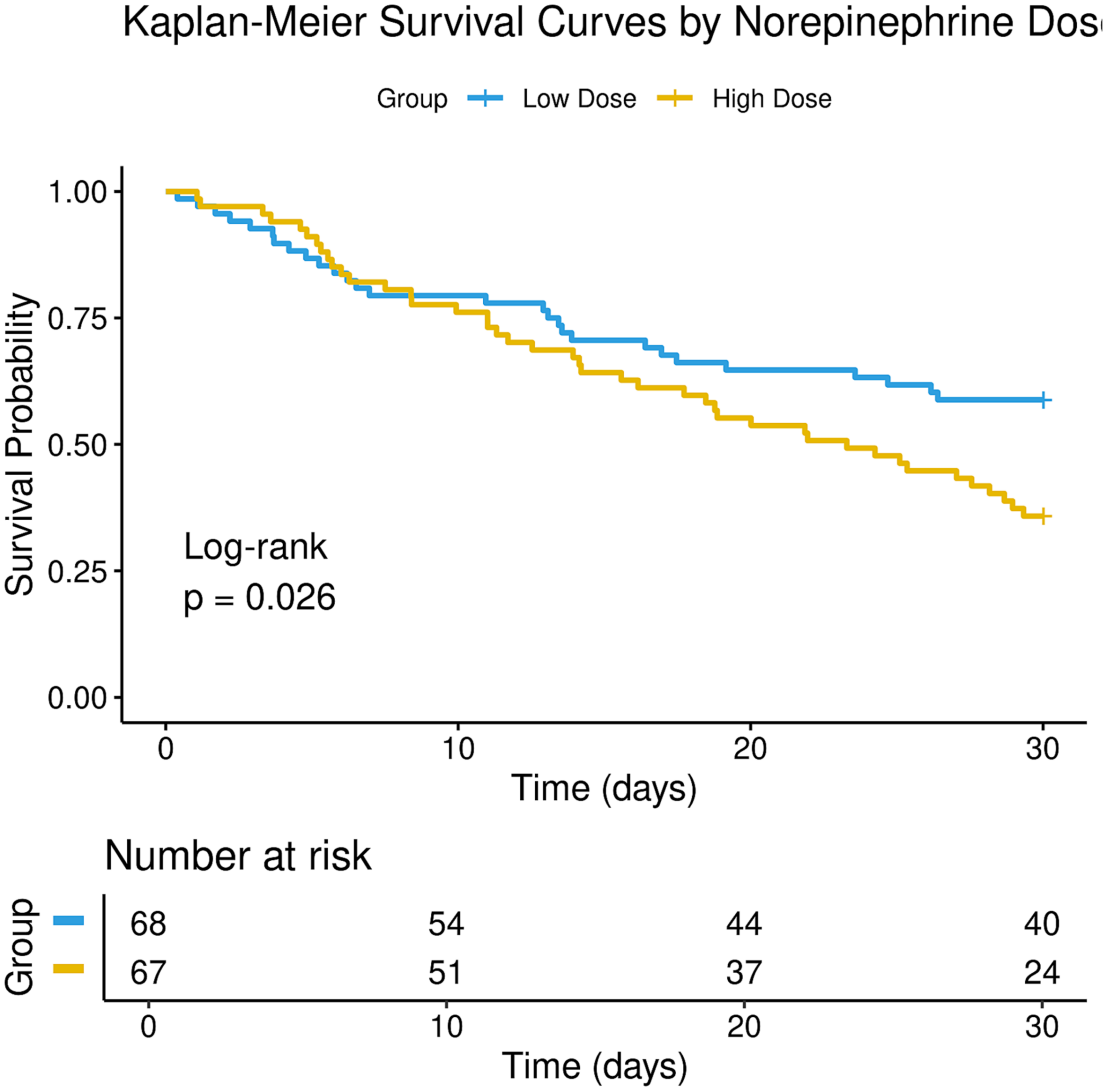
Kaplan–Meier survival curves.

Kaplan–Meier survival curves (Figure 2) revealed that the 30-day cumulative survival rate was significantly higher in the low-dose group compared to the high-dose group (64.7% vs. 38.8%, log-rank χ^2^ = 9.873, *p* = 0.002). In univariable Cox regression analysis, the high-dose group was associated with a significantly increased risk of 30-day mortality compared to the low-dose group (HR = 2.15, 95% CI 1.31–3.53, *p* = 0.002). To control for the influence of baseline imbalances, a multivariable Cox proportional hazards model was constructed (Table 2). After adjusting for age, baseline SOFA score, baseline lactate level, and primary diagnosis, assignment to the high-dose group remained an independent risk factor for 30-day mortality (adjusted HR = 1.92, 95% CI 1.14–3.24, *p* = 0.015). Furthermore, both the baseline SOFA score (adjusted HR = 1.18 per 1-point increase, 95% CI 1.07–1.30, *p* = 0.001) and the baseline lactate level (adjusted HR = 1.11 per 1 mmol/L increase, 95% CI 1.01–1.22, *p* = 0.032) were also independently associated with a higher risk of death (see Table 3).

**Table 2:** Comparison of secondary outcomes, tissue perfusion, and treatment metrics between groups.

Outcome measure	Low-dose group (*n* = 68)	High-dose group (*n* = 67)	Statistic	*P*-value
Secondary outcomes				
Achieved hemodynamic stability, n (%)	39 (57.35)	20 (29.85)	χ^2^ = 10.897	0.001
Successful ECMO weaning, n (%)	45 (66.18)	28 (41.79)	χ^2^ = 8.352	0.004
ICU LOS (days), mean ± SD	18.23 ± 9.45	22.87 ± 11.23	*t* = −2.647	0.009
Vasopressor support duration (h), mean ± SD	142.56 ± 68.34	198.73 ± 85.21	*t* = −4.278	<0.001
Tissue perfusion and organ function				
24-h lactate clearance (%), mean ± SD	18.34 ± 12.67	10.12 ± 15.43	*t* = 3.347	0.001
ΔSOFA72h (points), mean ± SD	−2.45 ± 1.89	−1.12 ± 2.34	*t* = −3.548	<0.001
Acute kidney injury, n (%)	25 (36.76)	36 (53.73)	χ^2^ = 4.012	0.045
New severe arrhythmia, n (%)	15 (22.06)	26 (38.81)	χ^2^ = 4.683	0.03
Treatment intensity metrics				
Mechanical ventilation days, mean ± SD	12.45 ± 7.89	14.78 ± 8.56	*t* = −1.634	0.104
Received CRRT, n (%)	22 (32.35)	34 (50.75)	χ^2^ = 4.723	0.03
Prbc transfused (u), median (IQR)	4.0 (2.0–8.0)	6.0 (4.0–12.0)	*Z* = −2.457	0.014

ICU LOS, intensive care unit length of stay; ΔSOFA72h, change in SOFA score from baseline to 72 h; CRRT, continuous renal replacement therapy; PRBC, packed red blood cells; IQR, interquartile range.

**Table 3:** Multivariable cox proportional hazards regression analysis for factors influencing 30-day survival.

Variable	β	SE	Wald χ^2^	Adjusted HR (95% CI)	*P*-value
High-dose group (vs. low-dose)	0.652	0.267	5.976	1.92 (1.14–3.24)	0.015
Age (per 1-year increase)	0.018	0.011	2.734	1.02 (0.998–1.04)	0.098
Baseline SOFA score (per 1-point increase)	0.165	0.051	10.582	1.18 (1.07–1.30)	0.001
Baseline lactate (per 1 mmol/L increase)	0.104	0.048	4.645	1.11 (1.01–1.22)	0.032
Primary diagnosis (ref: other)					
Acute myocardial infarction	0.312	0.301	1.076	1.37 (0.76–2.46)	0.299
Fulminant myocarditis	−0.287	0.389	0.543	0.75 (0.35–1.61)	0.461

HR, hazard ratio; CI, confidence interval; SOFA, sequential organ failure assessment; Ref, reference category.

### 3.3 Secondary outcomes and tissue perfusion indicators

**Figure 3:**
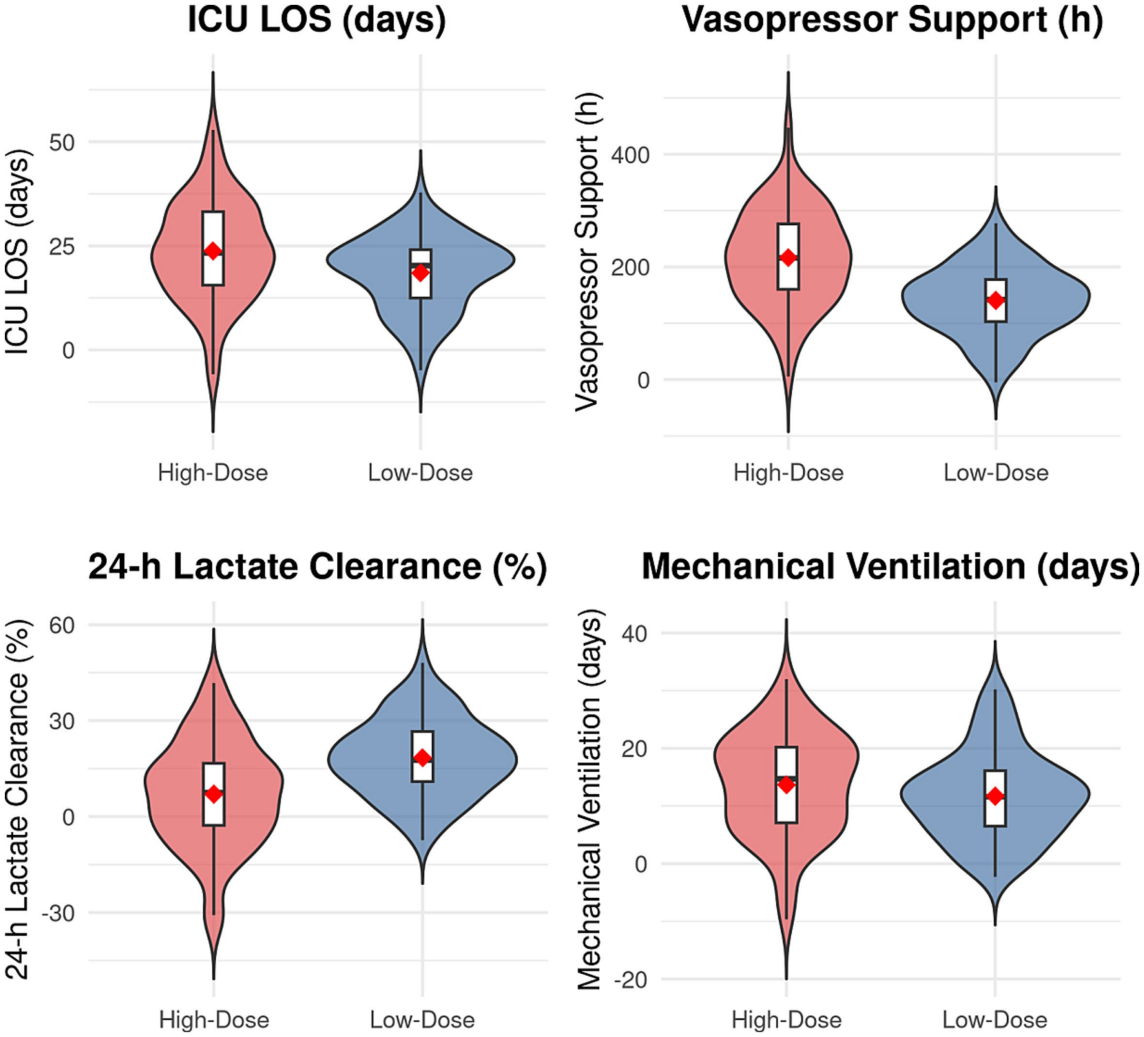
Clinical outcomes by treatment group.

Comparisons of secondary outcomes and treatment-related metrics between the two groups are presented in Table 3. The proportion of patients achieving the composite endpoint of hemodynamic stability was significantly lower in the high-dose group than in the low-dose group (29.9% vs. 57.4%, χ^2^ = 10.897, *p* = 0.001). The successful ECMO weaning rate was also significantly lower in the high-dose group (41.8% vs. 66.2%, χ^2^ = 8.352, *p* = 0.004). Furthermore, both the ICU length of stay and the total duration of vasopressor support were significantly prolonged in the high-dose group (*t* = 2.341, *p* = 0.021; *t* = 3.894, *p* < 0.001). Regarding tissue perfusion metrics, the 24 h lactate clearance rate was significantly lower in the high-dose group (*t* = −3.125, *p* = 0.002), and the degree of improvement in the SOFA score at 72 h post-ECMO initiation (ΔSOFA72h) was worse (*t* = 2.894, *p* = 0.004). The incidences of acute kidney injury and new-onset severe arrhythmias were both significantly higher in the high-dose group (χ^2^ = 4.012, *p* = 0.045; χ^2^ = 5.124, *p* = 0.024). In terms of treatment intensity, the proportion of patients requiring continuous renal replacement therapy was higher in the high-dose group (χ^2^ = 4.723, *p* = 0.030), and the total volume of packed red blood cells transfused during hospitalization was also greater (*t* = 2.567, *p* = 0.011) (see Table 2 and Figure 3).

### 3.4 Safety outcomes and complications

The comparison of safety outcomes and complication rates between the two groups is shown in Table 4. Patients in the high-dose group had a significantly increased risk of ECMO limb distal ischemia (16.4% vs. 4.4%, χ^2^ = 5.237, *p* = 0.022). Concurrently, the incidence of major bleeding events (BARC types 3–5) was also significantly higher in the high-dose group compared to the low-dose group (37.3% vs. 17.6%, χ^2^ = 6.782, *p* = 0.009). Regarding infectious complications, the rate of confirmed hospital-acquired infections was higher in the high-dose group, but the difference did not reach statistical significance (44.8% vs. 30.9%, χ^2^ = 2.874, *p* = 0.090) (see Table 4).

**Table 4:** Comparison of safety outcomes and complications between groups.

Complication	Low-dose group (*n* = 68)	High-dose group (*n* = 67)	χ^2^	*P*-value
ECMO limb distal ischemia, n (%)	3 (4.41)	11 (16.42)	5.237	0.022
Major bleeding event, n (%)	12 (17.65)	25 (37.31)	6.782	0.009
Confirmed hospital-acquired infection, n (%)	21 (30.88)	30 (44.78)	2.874	0.09

BARC, bleeding academic research consortium.

### 3.5 Sensitivity analysis and dose–response relationship

**Figure 4:**
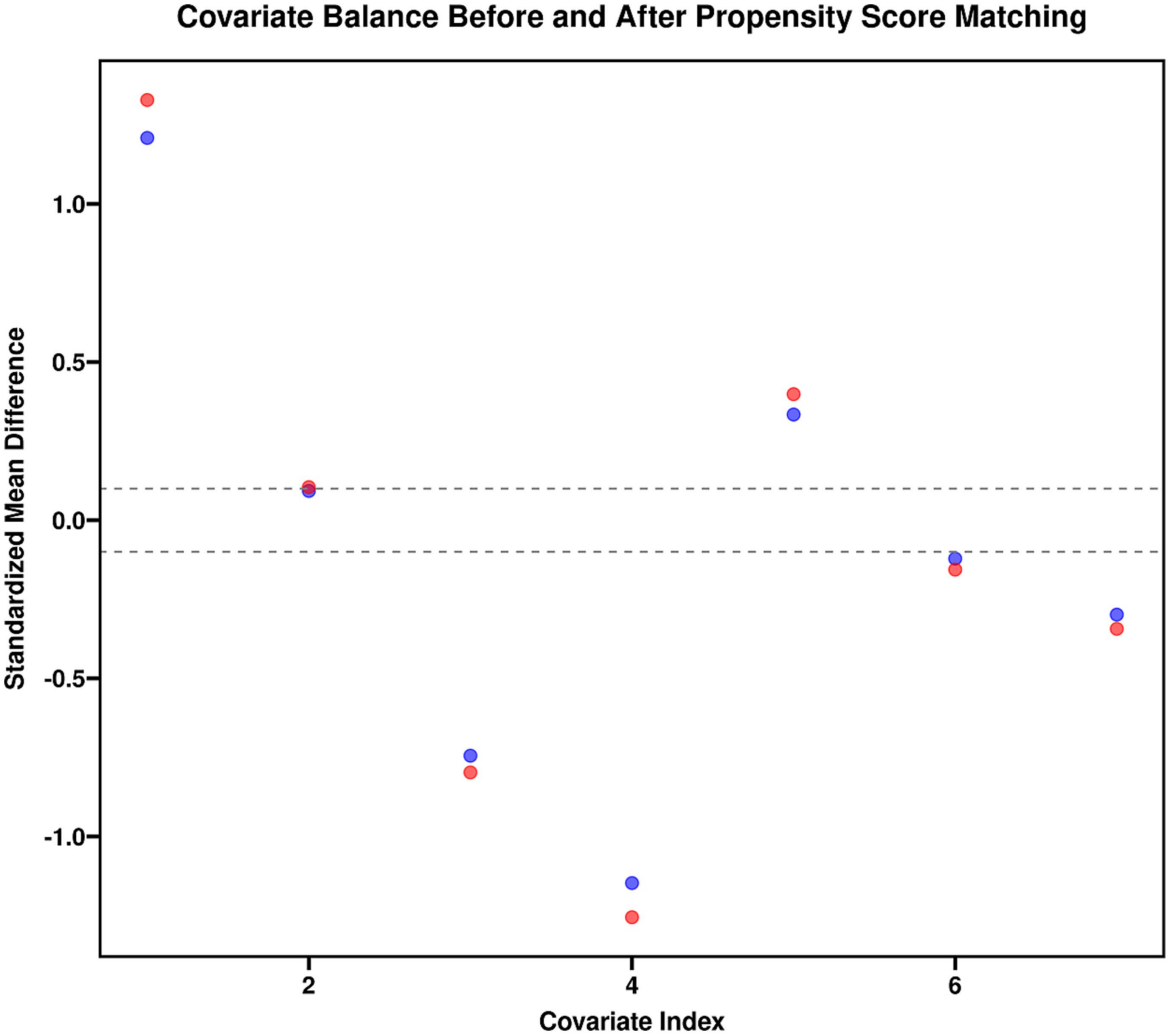
Covariate balance before and after propensity score matching.

To verify the robustness of the aforementioned results, propensity score matching was performed, successfully matching 52 patient pairs (104 patients). After matching, all baseline covariates included in the model achieved a good balance between the two groups (all standardized mean differences <0.1, *p* > 0.05). In the matched sample, the risk of 30-day mortality remained significantly higher in the high-dose group compared to the low-dose group (adjusted HR = 2.05, 95% CI 1.15–3.65, *p* = 0.015), consistent with the conclusion of the primary analysis (Figure 4).

**Figure 5:**
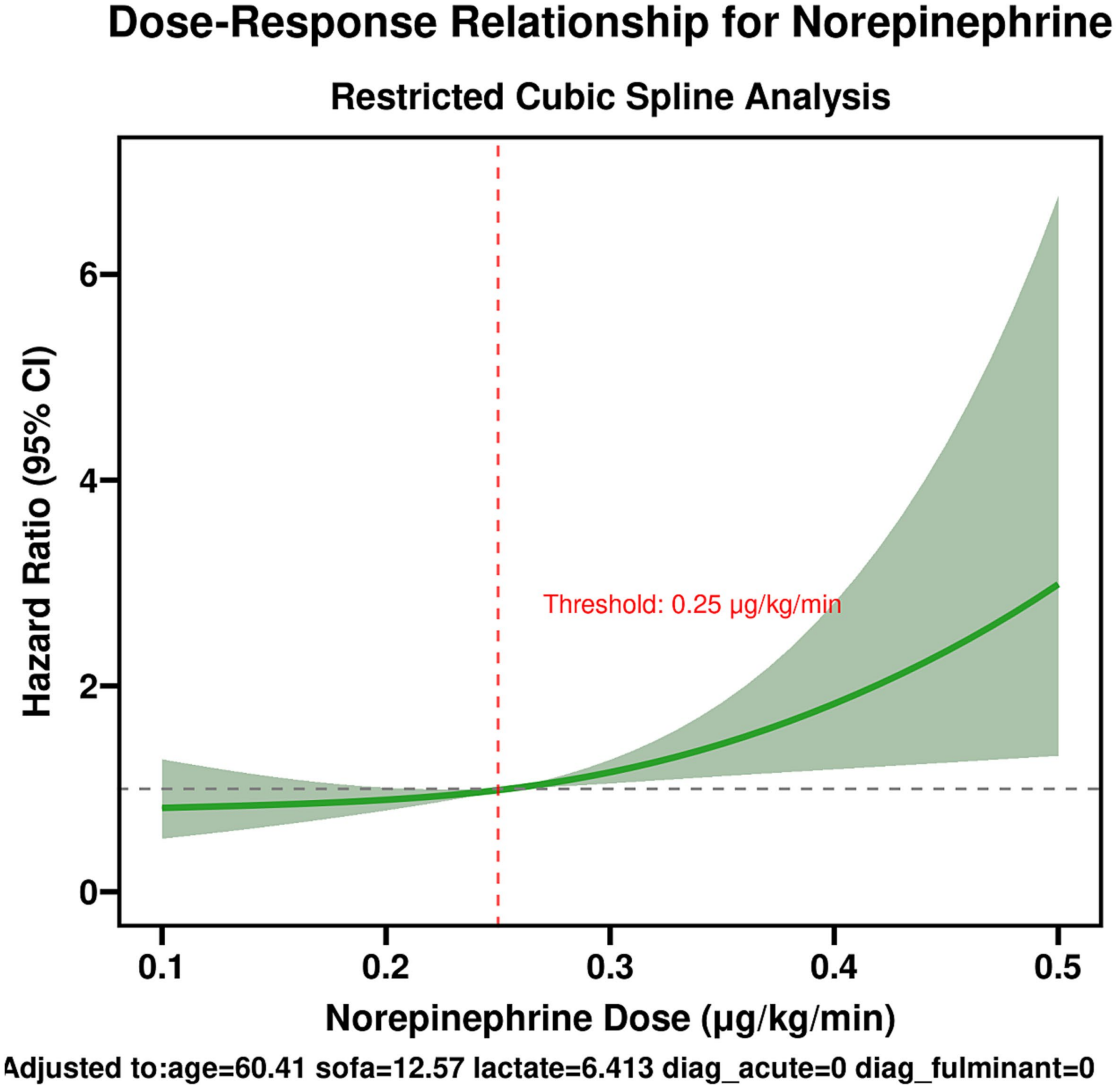
Dose–response relationship for norepinephrine.

Furthermore, a restricted cubic spline model was used to explore the potential non-linear relationship between the mean norepinephrine-equivalent dose (as a continuous variable) and the risk of 30-day mortality. The model demonstrated a monotonically increasing risk of death with escalating dose. Notably, the risk curve became steeper once the dose exceeded approximately 0.25 μg/kg/min (Figure 5). When the dose reached 0.40 μg/kg/min, the hazard ratio for mortality exceeded 2.5 compared to the reference point of 0.20 μg/kg/min. This result aligns with the conclusion drawn from the median-based group analysis.

### 3.6 Subgroup analysis

Prespecified subgroup analyses were performed according to baseline lactate level (≤6 mmol/L vs. >6 mmol/L) and primary diagnosis. Tests for interaction showed that the association between high-dose norepinephrine and increased mortality risk was consistent across lactate subgroups (P for interaction = 0.43) and diagnostic subgroups (P for interaction = 0.61), with no significant interactions observed. These findings suggest that, within the studied population, the mortality risk linked to high norepinephrine doses may represent a consistent effect across varying levels of tissue hypoperfusion and different underlying disease etiologies.

## 4 Discussion

This retrospective study sought to investigate the association between varying intensities of norepinephrine-equivalent dose during the initial phase of VA-ECMO support and clinical outcomes in patients with refractory shock. The central finding indicates that, although patients in the high-dose group presented with more severe baseline illness, a higher mean norepinephrine-equivalent dose during the first 72 h of ECMO support remained an independent predictor of increased 30-day mortality risk after adjustment for key confounders. This association was corroborated across multiple secondary outcomes, including inferior hemodynamic stability, poorer recovery of organ function, and a higher incidence of complications (16). Collectively, these results suggest that within the context of macrocirculatory support provided by ECMO, excessive use of vasopressor agents may not confer clinical benefit and could instead be linked to adverse outcomes (17).

Regarding Baseline Characteristics and Dose Stratification. In this study, patients in the high-dose group exhibited a more severe disease state even prior to ECMO initiation, including higher SOFA scores, lower mean arterial pressure, and elevated lactate levels. This baseline imbalance aptly reflects clinical reality: more critically ill patients often require more intensive vasopressor support to maintain perfusion (18). Although we performed statistical adjustment for this in our primary analysis using multivariable models, residual confounding from unmeasured factors may persist. Nevertheless, this highlights the importance of viewing vasopressor dose as a dynamic marker of illness severity rather than merely an intervention variable.

Regarding the Primary Survival Outcome. Multivariable Cox regression analysis confirmed that a higher norepinephrine-equivalent dose was an independent risk factor for 30-day all-cause mortality. This finding aligns with previous observations of a positive correlation between vasopressor dose and mortality in patients with shock (19). The underlying mechanisms may involve multiple pathways. High-dose norepinephrine may exacerbate intense *α*-adrenergic receptor-mediated vasoconstriction, potentially further impairing perfusion at the tissue level and aggravating organ ischemia in the setting of pre-existing systemic inflammation and microcirculatory dysfunction (20). Furthermore, the toxic effects of catecholamines—including direct myocardial injury, pro-arrhythmic effects, and immune dysregulation—may become more pronounced with high-dose exposure, thereby offsetting the circulatory support benefits conferred by ECMO.

Regarding Hemodynamic Stability and Organ Perfusion. The proportion of patients achieving the predefined composite endpoint of hemodynamic stability was significantly lower in the high-dose group, and lactate clearance was poorer. This indicates that merely elevating blood pressure to a specific range pharmacologically does not necessarily equate to improved effective oxygen delivery at the tissue level. Given that ECMO itself assumes a substantial portion of cardiac pumping and oxygenation functions, excessive peripheral vasoconstriction may increase afterload, potentially attenuating left ventricular unloading effects and even impeding microcirculatory recovery. Impaired lactate clearance directly reflects persistent tissue hypoxia and anaerobic metabolism, which is consistent with the worse evolution of the SOFA score and the higher incidence of acute kidney injury observed in the high-dose group (21). Prior research has also emphasized that successful shock resuscitation should focus on improving perfusion metrics rather than merely achieving target blood pressure numbers (22).

Regarding Treatment Intensity and Complications. Patients in the high-dose group demonstrated a higher treatment burden, evidenced by a longer duration of vasopressor use, a greater need for continuous renal replacement therapy, and more frequent packed red blood cell transfusions. These indicators collectively depict a more complex and protracted clinical course. The prolonged duration of vasopressor support is itself a marker of poor prognosis. The elevated risk of bleeding and transfusion may be related to tissue ischemia secondary to intensified vasoconstriction from high-dose norepinephrine, as well as potential underlying coagulopathy (23). These complications not only increase healthcare resource utilization but may also directly contribute to increased mortality risk, creating a vicious cycle.

Regarding ECMO Weaning and Length of Stay. The lower rate of successful ECMO weaning and the longer ICU stay in the high-dose group are indirect manifestations of their poorer prognosis. Successful liberation from ECMO depends on adequate recovery of cardiac function and systemic circulation. A sustained requirement for high-dose vasopressors may indicate insufficient recovery of native cardiac function or the development of severe complications (e.g., severe sepsis, right heart failure), rendering patients unable to be weaned from circulatory support (24). The prolonged ICU stay comprehensively reflects the severity of illness, the occurrence of multiple complications, and a slow recovery trajectory.

Regarding Safety Endpoints. The incidences of new-onset severe arrhythmias and limb ischemia were higher in the high-dose group. The β1-adrenergic receptor agonist effect of norepinephrine may become more pronounced at higher doses, directly increasing myocardial oxygen demand and facilitating the onset of arrhythmias (1). Limb ischemia is a known complication of VA-ECMO compounded by intense peripheral vasoconstriction, for which high-dose vasopressor use is undoubtedly a significant contributing factor. These safety issues directly impact patients’ chances of survival and functional outcomes.

This study has several limitations. First, the retrospective, single-center design is inevitably susceptible to selection bias and confounding, although we employed multivariable adjustment and propensity score matching as sensitivity analyses. Second, the norepinephrine dose was averaged over the first 72 h, failing to capture the potential association between its dynamic trajectory and outcomes. Third, the conversion formulas used to convert different vasoactive agents (epinephrine, dopamine, and vasopressin) to norepinephrine-equivalent doses, while based on routine clinical practice and widely adopted in previous studies, are not derived from a universally accepted gold-standard conversion system. This methodological choice may introduce some degree of dose estimation error and represents an inherent limitation of this study. Fourth, while we focused primarily on drug dose, we did not conduct an in-depth analysis of specific details of hemodynamic management, such as precise mean arterial pressure targets or fluid management strategies, which are also critical factors influencing prognosis. Future prospective studies are needed to explore perfusion-oriented hemodynamic management strategies that minimize vasopressor use during ECMO support and to define their optimal target ranges.

In conclusion, for patients receiving VA-ECMO support for refractory shock, a higher norepinephrine-equivalent dose during the initial period of ECMO operation is independently associated with increased 30-day mortality, poorer recovery of organ function, and a higher risk of complications. Despite the confounding influence of greater baseline illness severity, this association remained robust after adjustment. Clinicians utilizing ECMO for circulatory support should carefully evaluate the necessity of vasopressor agents, striving to achieve a balance between maintaining essential perfusion pressure and avoiding the potential harms associated with high-dose catecholamines. Our findings suggest that several objective parameters may help guide this balance: a mean norepinephrine-equivalent dose exceeding 0.28 μg/kg/min (the median value in our cohort) during the first 72 h was associated with significantly worse outcomes. Additionally, persistently elevated lactate levels with a 24 h clearance rate <10%, or a worsening or stagnant SOFA score at 72 h post-ECMO initiation, may serve as objective indicators of heightened risk, prompting clinicians to reconsider vasopressor intensity, evaluate for unrecognized hypoperfusion, or consider escalation of mechanical circulatory support or adjunctive therapies. These parameters could be integrated into clinical decision-making algorithms, though prospective validation is needed.

## Data Availability

The datasets presented in this article are not readily available because the datasets generated during this study are available from the corresponding author upon reasonable request, subject to restrictions imposed by the approving ethics committee, institutional data governance policies, and patient privacy protections. Access requires a formal proposal and the execution of a data use agreement stipulating that the data will be used only for the specified research purpose, will not be redistributed, and will be securely protected. Availability of specific data may be further limited to prevent any potential compromise of participant anonymity. Requests to access the datasets should be directed to Zhenzhen Chen; H303587670@163.com.
